# Preparation of the Mouth Prior to Making an Artificial Denture

**Published:** 1915-06

**Authors:** Robert Brown, Charles P. Bower


### PREPARATION OF THE MOUTH PRIOR TO MAKING AN ARTIFICIAL DENTURE.

READ BEFORE THE STUDENTS' DENTAL SOCIETY OF THE UNIVERSITY OF MICHIGAN, MARCH 29, 1915.

BY ROBERT BROWN AND CHARLES P. BOWER.

There always has been a need of artificial dentures and always will be. Every dentist owes to his patients duties along this line as well as other departments of dental practice. Plate work is treated slightingly by many dentists, principally, because they never learn to do this kind of work well enough to get interested in it. One of the most important factors in the work is that they do not master the fundamentals. One of the fundamentals is the careful study of the conditions as they find them. Another is the ability to plan an adequate procedure for individual cases.

Upon the presentation of the patient and request for an artificial denture, the first duty on the part of the dentist is to make a careful and complete diagnosis of the mouth, both with and without the aid of the mouth mirror. This diagnosis should not be hurriedly made as is too often the case. No two cases are alike and no rule will apply to all. The examination is two-fold in its purpose. First we must make a careful estimate of the general hygienic conditions of the oral tissues in general, but especially of those involved directly in the proper denture. Secondly, we should study the tissues to ascertain what support they will afford for the denture. Under the first head, the gums may be hypertrophied, inflamed, or infected. There may be gingivitis, pyorrhea, ulcerations, tumors, or eruptions from specific diseases. Obviously, any of such conditions must be removed before attempting the placement of any kind of a denture. To remove these conditions, we must know the source of the disease, and if practicable, remove the cause. All this necessitates, first, a thorough preliminary cleansing of the mouth. This should be done to obtain a clear and conclusive diagnosis and to secure accurate final judgment. This is easily accomplished in most cases by mouth irrigation with slightly medicated water and where there is infection, or serious contamination present, with stronger chemical disinfectants, as hot soda water or acidulated water for carbohydrates and fermentation deposits. In case of infections, the mount may be washed with dilute peroxide of hydrogen. In partial cases, teeth having deposits of calculus, the teeth should be surgically cleaned. All teeth that are present, other than those which are to be extracted, should be cleaned and polished before impressions are attempted. If there are teeth to be extracted, we should proceed on well defined methods and for good reasons. We should, of course, extract diseased teeth that are incurable, likewise all irritable teeth that can not be made comfortable, or such as may defeat all well planned methods of securing proper functions. We may also extract teeth badly affected with peridentitis, badly deformed, malposed, or discolored teeth. As well as sound teeth which in many cases can not be made artistic, roots not needed for anchorage or contour, and which are, or may become unhealthy; also any teeth that interfere with a proper technical treatment of the case. Good teeth should be preserved when necessary for retention. Lower dentures often need clasp support and isolated teeth, preferably the cuspids, should be retained for such purposes. Oftimes it may be advisable to crown a root for clasp support. This can be done to good advantage in many cases. If teeth have been lately extracted the condition of the gums should be noted for comfort to the patient, and for sanitary reasons. No denture should be placed in the mouth until the gums have completely healed and closed in well over the arch. This may take from two to three weeks, dependent on the age and physical condition of the patient. A temporary denture can be made very soon after extraction, but a permanent denture should not be made until a certain amount of resorption of the alveolar process has taken place. This varies in different individuals, from three months to a year. Before this, a temporary denture should be made to prevent excessive resorption and for the comfort of the patient. In old persons, or those who have worn dentures for any length of time, an examination of the mucous and sub-mucous tissues should be made by passing the finger over the entire surface of the mouth that is to be used by the denture, to discover the hard and soft areas of tissue. All these should be carefully noted and allowances made for same, in making the denture, especially in selecting the impression materials to be used, and the technique to be followed in taking the impression and treatment of the models afterwards. Some other points that need to be noted are: the size of areas of the maxillary and mandibular surfaces to be utilized and any peculiarity as to the muscle or ligamentary attachments that may be encroached upon to the detriment of close adaption or plate adhesion, such as the frenum in the upper at the incisal attachment and the attachment of the genio hyoglossal muscle. These may be so strong and prominent in some cases as to necessitate surgical treatment in order to make the properly adjusted denture. The salivary and mucous secretions should also be noted as they often play important parts in the denture retention. A slimy or thick mucous, ropey secretion is not favorable, and in such a case, systemic treatment may be advised before the denture is finally inserted. We should always keep in mind the value of a properly constructed denture from the functional standpoint, and the possible menace to good health of a poorly constructed one.

We may rest assured that it will be accidental only if success shall come from a routine procedure in technical construction based upon a careless or unscientific preliminary consideration of the peculiar conditions of every individual case that falls to our lot to treat. We can not always complete our diagnosis by the most thorough oral examination we can make of the patient's mouth. It may be necessary in some cases to wait until we have taken the impressions and bite and properly mounted them and after careful inspection feel that our diagnosis is sufficiently clear to warrant a final decision as to the exact character of the denture best suited to the patient.

We make a plea for the most painstaking and comprehensive diagnosis possible as the one, first, and most important procedure in the successful construction of prosthetic substitutes of every sort.

